# Presence of Cholesterol in Non-Animal Organisms: Identification and Quantification of Cholesterol in Crude Seed Oil from *Perilla frutescens* and Dehydrated *Pyropia tenera*

**DOI:** 10.3390/molecules26123767

**Published:** 2021-06-21

**Authors:** Min-Ji Oh, Hee-Jin So, Eun-Sik Hong, Jung-Ah Shin, Ki-Teak Lee

**Affiliations:** 1 Department of Food Science and Technology, Chungnam National University, 99 Daehak-ro, Yuseong-gu, Daejeon 34134, Korea; mjoh@o.cnu.ac.kr (M.-J.O.); waigtd@o.cnu.ac.kr (H.-J.S.); hes9730@o.cnu.ac.kr (E.-S.H.); 2 Department of Food Processing and Distribution, Gangneung-Wonju National University, 7 Jukheon-gil, Gangneung 25457, Gangwon-Do, Korea; jashin@gwnu.ac.kr

**Keywords:** cholesterol, algae, perilla seed oil, lathosterol, GC-FID, HPLC-ELSD, LC-MS/MS

## Abstract

Studies have reported that cholesterol, a molecule found mainly in animals, is also present in some plants and algae. This study aimed to determine whether cholesterol exists in three dehydrated algae species, namely, *Pyropia tenera, Saccharina japonica,* and *Undaria pinnatifida*, and in one plant species, namely, *Perilla frutescens* (four perilla seed oil samples were analyzed). These species were chosen for investigation because they are common ingredients in East Asian cuisine. Gas chromatography-flame ionization detection (GC-FID) analysis found that cholesterol was present in *P. tenera* (14.6 mg/100 g) and in all four perilla seed oil samples (0.3–0.5 mg/100 g). High-performance liquid chromatography with evaporative light-scattering detection (HPLC-ELSD) also demonstrated that cholesterol was present in *P. tenera* (14.2 mg/100 g) and allowed the separation of cholesterol from its isomer lathosterol. However, cholesterol could not be detected by HPLC-ELSD in the perilla seed oil samples, most likely because it is only present in trace amounts. Moreover, liquid chromatography-tandem mass spectrometry (LC-MS/MS) confirmed the presence of cholesterol in both *P. tenera* and perilla seed oil. MRM results further suggested that lathosterol (a precursor of cholesterol) was present in *P. tenera.*

## 1. Introduction

Cholesterol is an unsaturated alcohol belonging to the steroid family of compounds [1,2], whose amphiphilic character, imparted by the hydroxyl group (OH), helps to regulate the membrane fluidity of cells [3,4]. It does so by forming hydrogen bonds with water molecules and other lipid components of the cell membrane [5,6]. In the human body, cholesterol is biosynthesized in the liver and is required for the formation of vitamin D and steroid hormones [7,8,9].

In order for the body’s cholesterol level to be maintained at a constant level, the amount of cholesterol synthesized by the liver varies depending on food intake [10,11]. When dietary cholesterol intake increases, this molecule accumulates on the inner walls of arteries, and its increased lipid concentration in the blood can cause cardiovascular diseases such as atherosclerosis and hyperlipidemia [12]. Despite a lack of evidence confirming this link between dietary cholesterol intake, blood vessel cholesterol levels, and risk of heart disease, it is nonetheless generally recommended that cholesterol intake is kept below 300 mg/day [13]. For this reason, cholesterol content is required to be shown on nutrition facts labels and should only be marked as zero when its content is less than 2 mg/100 g of food [14]. Previous studies investigating the presence of cholesterol in vegetable oils and algae have shown conflicting results, with some studies finding no evidence of cholesterol in either. While some studies have been unable to detect cholesterol in vegetable oils (e.g., perilla oil, basil seed oil, and flaxseed oil) [15] or red algae (e.g., *Halymenia dilatata*) [16], several other studies have suggested that small amounts are present in vegetable oils (e.g., sesame oil, corn oil, rapeseed oil, and camelina oil) [17] and algae [18,19]. For example, Govindan et al. [18] detected cholesterol in 11 species of Caribbean marine algae, and Sánchez-Machado et al. [19] found cholesterol in two species of red algae (*Porphyra* sp. and *Palmaria* sp.). In addition to vegetable oils and algae, cholesterol has also been detected in plants [20,21]. Thus, to improve our understanding of cholesterol presence in non-animal organisms, it is necessary to clarify these contradictory findings.

Some studies have detected both lathosterol and cholesterol in plants and algae [16,22]. For example, Eller et al. [22] reported that both molecules were present in tomato seed oil. Meanwhile, Maheswari et al. [16] detected only lathosterol, a biosynthetic precursor of cholesterol, in red algae (*Halymenia dilatata*). Both compounds are structural isomers (C_27_H_46_O, molecular weight = 386.65 g/mol), with different double bond positions. The double bond in lathosterol is located between carbons 7 and 8, whereas in cholesterol it is located between carbons 5 and 6.

**Figure 1:**
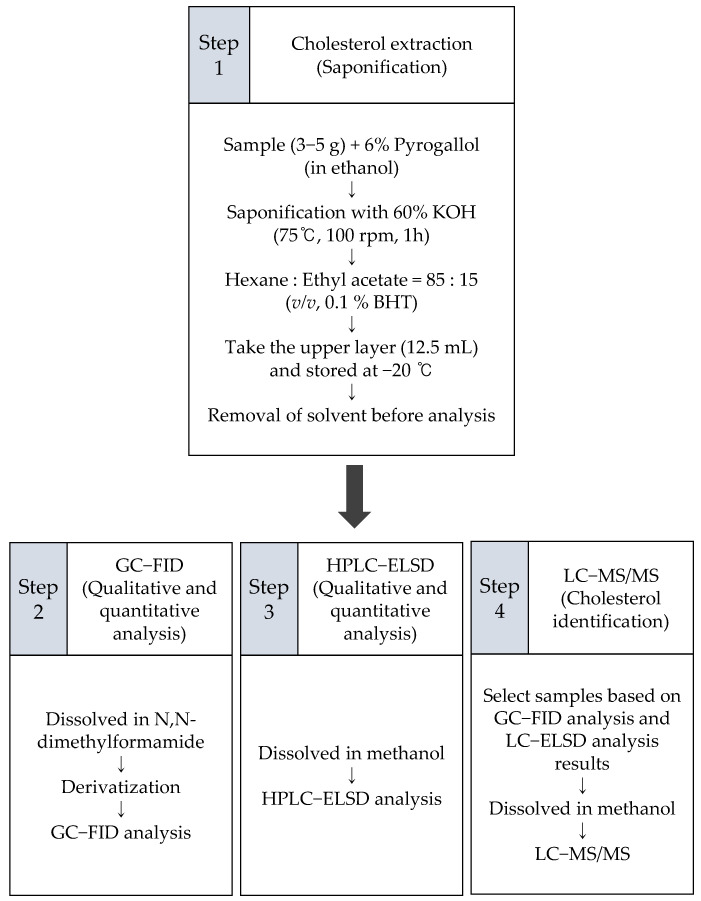
Scheme of the experimental design.

Building on the work carried out in the studies mentioned above [17,18,19,20,21,22], this work aims to use three analytical methods to determine the levels of cholesterol in non-animal organisms, particularly, algae and perilla seed oil (categorized as a vegetable oil). To our knowledge, this is the first study to quantify cholesterol content in perilla seed oil. Red algae (*Pyropia tenera*), brown algae (*Saccharina japonica* and *Undaria pinnatifida*), and perilla seed oil (i.e., crude oil extracted from *Perilla frutescens*) were selected for this study because they are common ingredients in East Asian cuisine (particularly in Korea and Japan). Gas chromatography with flame ionization detection (GC-FID), high-performance liquid chromatography with evaporative light-scattering detection (HPLC-ELSD), and liquid chromatography-tandem mass spectrometry (LC-MS/MS) were employed according to the experimental scheme below (Figure 1). Based on the previous results [17,18,19,20,21,22], little content was expected if cholesterol exists in algae and perilla seed oil, meaning that the possible presence of its isomer lathosterol could mislead the results depending on the characteristics of the analytical methods. The results from these three analytical methods are presented below, and cholesterol content is reported for samples in which its presence was confirmed.

## 2. Results and Discussion

### 2.1. Determination of Cholesterol by Gas Chromatography (GC)

GC is a technique widely used in cholesterol analysis, whereby separation is achieved depending on the volatility of the analytes and the chemical and/or physical interaction between the analytes and the stationary phase of the column. The HP-5 column used in this study has been widely used for sterol analysis and contains a 5% phenyl-methylpolysiloxane copolymer stationary phase [23].

**Figure 2:**
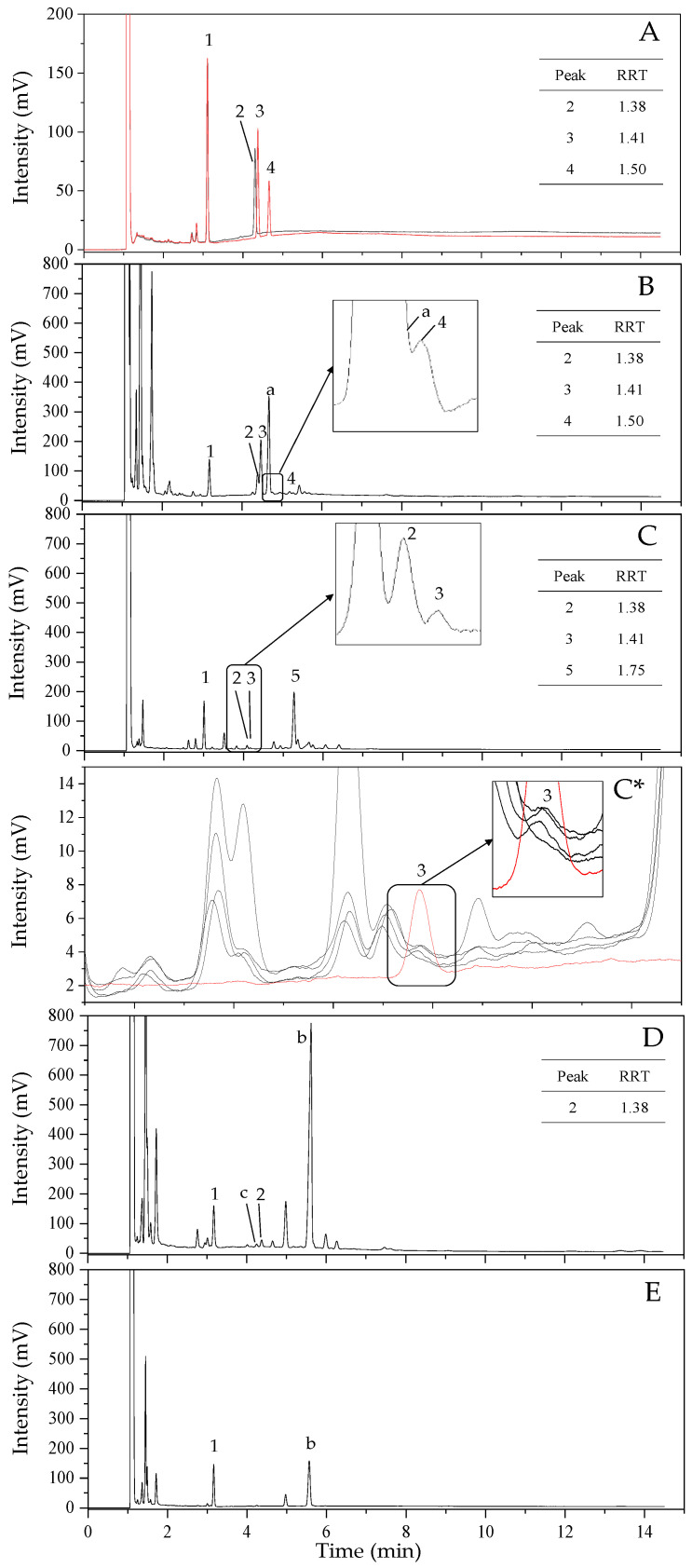
Gas chromatograph-flame ionization detector (GC-FID) chromatogram of a mixed standard (cholesterol, lathosterol, and α-tocopherol) and samples. (**A**), mixed standard; (**B**), *Pyropia tenera*; (**C**), perilla seed oil; (C *), cholesterol peak of four perilla seed oil samples; (**D**), *Saccharina japonica*; (**E**), *Undaria pinnatifida.* Peak identification: 1, 5-α-cholestane (IS); 2, α-tocopherol; 3, cholesterol; 4, lathosterol; 5, β-sitosterol a-b, unknown peak; c, unknown peak (RRT: 1.35) around lathosterol and cholesterol. Relative retention time (RRT): value obtained by dividing the compound retention time by the internal standard retention time.

Figure 2A shows that a mixed standard containing cholesterol and lathosterol was efficiently separated at 4.37 min and 4.65 min, corresponding to a relative retention time (RRT) of 1.41 and 1.5, respectively. The boiling points of cholesterol and lathosterol (i.e., isomers with a double bond in different positions) are known to be 480 ± 14 °C (at 760 mmHg) [24] and 479.5 ± 44 °C (at 760 mmHg) [25], respectively, while the log *p* values are 9.85 and 9.76, respectively. Even though there are no distinct differences in physiochemical properties that would allow the separation of the two isomers, efficient separation was achieved by GC in this study. An additional peak to the left of the cholesterol peak on the GC chromatogram was identified as α-tocopherol (peak 2 in Figure 2A) by spiking the standard.

From the GC analysis, a cholesterol peak was observed in the *P. tenera* sample (peak 3 in Figure 2B), with an RRT of 1.41. Previous studies have reported a higher occurrence of cholesterol in red algae than in brown algae. According to Kamenarska et al. [26], cholesterol accounted for 59.8−96.2% of the total sterol fraction in 10 species of red algae (*Bangia fuscopurpurea, Corallina mediterranea, Corallina granifera, Callithamnion granulatum, Ceramium elegans, Ceramium rubrum, Polysiphonia denudate, Polysiphonia denudate f. fragilis, L. coronopus,* and *L. papillosa*). In addition, Sánchez-Machado et al. [19] reported that cholesterol accounted for 1.3–8.6% of the sterols analyzed in two species of red algae (*Porphyra* sp. and *Palmaria* sp.), whereas it was either not detected or only detected in trace quantities in the five species of brown algae studied. Furthermore, Patterson [27] reported that in 37 species of red algae, the major sterol present was cholesterol; however, in 36 brown algae species, the major sterol present was fucosterol. In contrast, Govindan et al. [18] reported that 11 species of Caribbean marine algae, including red, brown, and green algae species, contained cholesterol. Cholesterol was detected in the range of 0.40–1.22 μg/mg of crude extract in red algae species (*Acanthophora spicifera, Laurencia papillosa, Galaxaura oblongata, Liagora species,* and *Gracilaria foliifera*). Levels of 0.54–0.58 and 0.06–0.64 μg/mg were detected in brown algae species (*Lobophora variegate* and *Sargassum polyceratium*) and green algae species (*Bryopsis plumosa, Caulerpa racemosa, C. sertulariodes,* and *Ulva lactuca*)*,* respectively [18].

To quantify the cholesterol content of *P. tenera*, a calibration curve (y = 9.5761x + 0.0266, *R^2^* = 0.9989) was used. From this, the cholesterol content of *P. tenera* was found to be 14.6 mg/100 g dried weight (Table 1). Under the assumption that the FID responses (involving the combustion of organic samples) of cholesterol and lathosterol ions are similar, the content of lathosterol was determined to be above the LOQ of cholesterol (peak 4 in Figure 2B). A cholesterol peak was not detected in the GC-FID chromatograms obtained for either brown algae species studied (*S. japonica* and *U. pinnatifida*) (Table 1, Figure 2D,E). In summary, cholesterol was found to be present in detectable amounts in the red algae species (*P. tenera*), and although it was undetectable in the brown algae studied here, a wider diversity of species would be necessary for future investigation. In addition, lathosterol was not observed in brown algae (Figure 2D,E).

Ghaleshahi et al. [15] were unable to detect cholesterol in vegetable oils (i.e., perilla oil, basil seed oil, and flax seed oil). Similarly, Watanabe et al. [28] were unable to detect cholesterol in canola oil or perilla oil. In contrast, Torri et al. [29] reported that cholesterol (0.15% among the sterols) was found in perilla oil. Similarly, a previous study also showed that cholesterol was present in 20 vegetable oils including perilla oil [30]. In the present study, cholesterol (RRT = 1.41) was detected by GC in all four samples of perilla seed oil (Figure 2C *), quantified as 0.3−0.5 mg/100 g (Table 1). Lathosterol, however, was not observed in any of the perilla seed oil samples.

In addition to the cholesterol and lathosterol peaks, the GC chromatograms of perilla seed oil displayed peaks corresponding to other sterols, the largest of which being peak 5 (Figure 2C), which was identified as β-sitosterol by use of a standard. Although further identification was not performed, peak a (Figure 2B) and peak b (Figure 2D,E) were assumed to be desmosterol and fucosterol, based on their previously reported high concentrations in red and brown algae, respectively [19,27].

### 2.2. Determination of Cholesterol by High-Performance Liquid Chromatography (HPLC)

As mentioned earlier, several studies have shown controversial results regarding the presence or the levels of cholesterol in algae and plants; however, this study has confirmed its presence in *P. tenera* and perilla seed oil by GC analysis. Therefore, the same sample used in the GC analysis was also used to examine the presence of cholesterol via HPLC-ELSD. Optimized HPLC conditions were necessary for efficient separation, considering the structural similarity of cholesterol and lathosterol. One study found that only one out of 19 different column types proved effective for the efficient separation of the two isomers [31], indicating the importance of selecting an appropriate column. A Poroshell 120 EC-C18 column was used in this study, with an end-capped octadecylsilane stationary phase. End-capping offers the advantage of increasing the retention of hydrophobic compounds [32].

**Figure 3:**
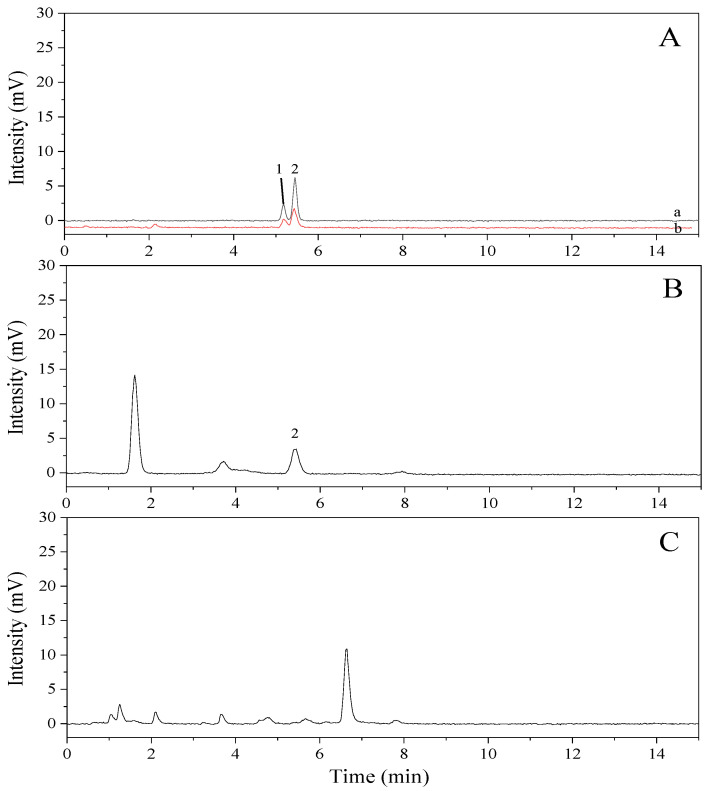
Chromatograms of mixed standards and samples using high-performance liquid chromatography-evaporative light-scattering detection (HPLC-ELSD). (**A**), mixed standard (a, lathosterol standard 0.005 mg/mL and cholesterol standard 0.01 mg/mL; b, lathosterol standard 0.0025 mg/mL and cholesterol standard 0.005 mg/mL). (**B**), *Pyropia tenera*; (**C**), perilla seed oil. Peak identification: 1, lathosterol; 2, cholesterol.

Cholesterol content in the samples was quantified under these optimized chromatographic conditions, and the results were compared with those from the GC analysis. Cholesterol quantification was performed with ELSD, which proceeds by evaporating off the mobile phase and using nitrogen gas to pass the sample through a heated drift tube before the remaining analyte particles collide with the laser of the detector. The cholesterol content of *P. tenera* was found to be 14.2 mg/100 g (Table 1), which was similar to the value determined by GC (Figure 3B). Meanwhile, as shown by the GC results, cholesterol was not detected in *S. japonica* and *U. pinnatifida* using HPLC-ELSD (Table 1). The calibration curve used for the determination of cholesterol content was y = 0.9376x + 0.0866 (*R^2^* = 0.9985), and the LOD and LOQ were 0.23 and 0.66 mg/100 g, respectively (Table 1). Unlike the GC results, however, lathosterol was not observed in *P. tenera* using HPLC-ELSD (Figure 3B). This discrepancy can be attributed to the different characteristics of the two analytical methods. In the case of perilla seed oil, peak assignment for cholesterol and lathosterol was not possible (Figure 3C) because the resolution was too low. Therefore, further analysis was performed by LC-MS/MS in order to confirm the presence of cholesterol in *P. tenera* and perilla seed oil. 

### 2.3. Identification of Cholesterol by Liquid Chromatography-Tandem Mass Spectrometry (LC-MS/MS)

Each sample of *P. tenera* and perilla seed oil in which cholesterol was found to be present by GC and HPLC-ELSD analysis was further analyzed by LC-MS/MS, which has a greater level of accuracy on the molecular level. In this study, MS/MS was used with atmospheric pressure chemical ionization (APCI), whereby the analyte is sprayed from a capillary tube before being vaporized at a high temperature and ionized in an electron beam [33,34]. APCI is a suitable method for analyzing compounds with low polarity and low molecular weight [35,36]. In addition, APCI is an efficient method for analyzing cholesterol because an additional derivatization step is not required. However, this step may be necessary when analyzing neutral and non-polar compounds (e.g., such as sterols) by electrospray, as they are otherwise not well ionized [35,37]. 

**Figure 4:**
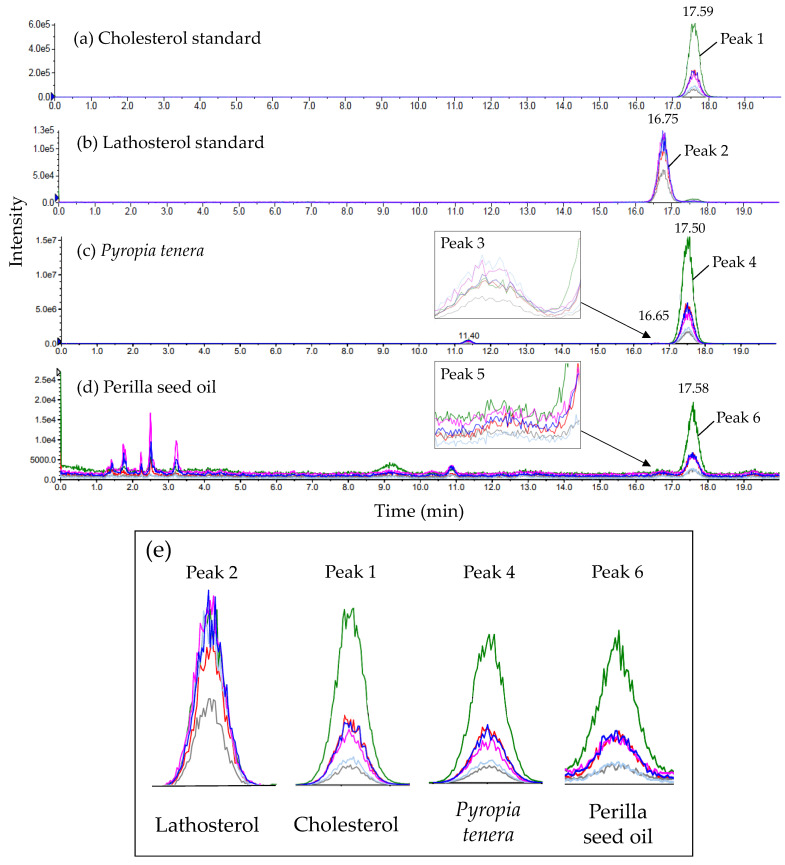
Multiple reaction monitoring (MRM) spectra of cholesterol standard (**a**), lathosterol standard (**b**), *Pyropia tenera* (**c**), perilla seed oil (**d**). Enlarged MRM spectrum of each peak 1, 2, 4, and 6 (**e**).

Cholesterol and its isomer lathosterol often exhibit identical ion fragments in LC-MS/MS because of their structural similarity and identical molecular weights (386.5 g/mol) [38]. It was therefore necessary to develop an appropriate chromatographic separation method for the two compounds. Even though the two compounds were efficiently separated using a Poroshell 120 EC-C18 column as mentioned earlier, separation by LC-MS/MS was also performed to verify the peak assigned to cholesterol in the *P. tenera* and perilla seed oil samples. According to LC-MS/MS with multiple reaction monitoring (MRM) mode in this study (Table 2), the precursor ion mass of each standard of cholesterol (peak 1 in Figure 4 = 17.59 min) and lathosterol (peak 2 in Figure 4 = 16.75 min) was 369.5 *m/z*, which is the value of removed H_2_O located in carbon 3 of cholesterol and lathosterol, and the H^+^ combined value. In addition, both isomers were found to have identical product ions (81, 95, 147, 161.1, 189.1, and 257.1 *m*/*z*), whereas their MRM patterns were different (Figure 4). To distinguish the isomers from one another, the ratio of peak area to smallest peak area was obtained for each product ion. In Table 2, the cholesterol fragmentation pattern is represented by the peak area of each product ion expressed relative to the product ion with the smallest peak area (257.1 *m*/*z*). The relative ratios were 3.3 (81 *m*/*z*), 2.7 (95 *m*/*z*), 3.4 (147 *m*/*z*), 9.4 (161.1 *m*/*z*), and 1.4 (189.1 *m*/*z*), respectively. In the case of lathosterol, the relative peak area ratios of the product ions to the product ion with the smallest peak area (189.1 *m*/*z*) were 5.8 (81 *m*/*z*), 7.0 (95 *m*/*z*), 10.7 (147 *m*/*z*), 43.2 (161.1 *m*/*z*), and 2.6 (257.1 *m*/*z*), respectively. For the *P. tenera* sample (Figure 4), peak 4 (RT = 17.50 min) was assigned to cholesterol and exhibited a relative ratio pattern—where relative ratio refers again to the ratio of each remaining product ion [(i.e., 3.2 (81 *m*/*z*), 2.5 (95 *m*/*z*), 3.3 (147 *m*/*z*), 9.2 (161.1 *m*/*z*), and 1.4 (189.1 *m*/*z*)] to 257.1 *m*/*z* (the smallest peak area)—which was comparable with that of the cholesterol standard. For peak 6 (RT = 17.58 min) of the perilla seed oil, 257.1 *m*/*z* was the product ion with the smallest peak area, and the relative ratios were 3.4 (81 *m*/*z*), 2.7 (95 *m*/*z*), 3.4 (147 *m*/*z*), 10.0 (161.1 *m*/*z*), and 1.4 (189.1 *m*/*z*). Therefore, from the fragmentation pattern determined by MRM, the corresponding peaks in *P. tenera* and perilla seed oil were confirmed as cholesterol.

Furthermore, the MRM results in Figure 4 indicate that peak 3 in the spectrum of *P. tenera* should be lathosterol, as the fragmentation pattern and retention time correspond to those of the lathosterol standard. In contrast, the peak expected for lathosterol in perilla seed oil was not clearly observed in this study, most likely due to lathosterol quantities being below the LOD.

**Figure 5:**
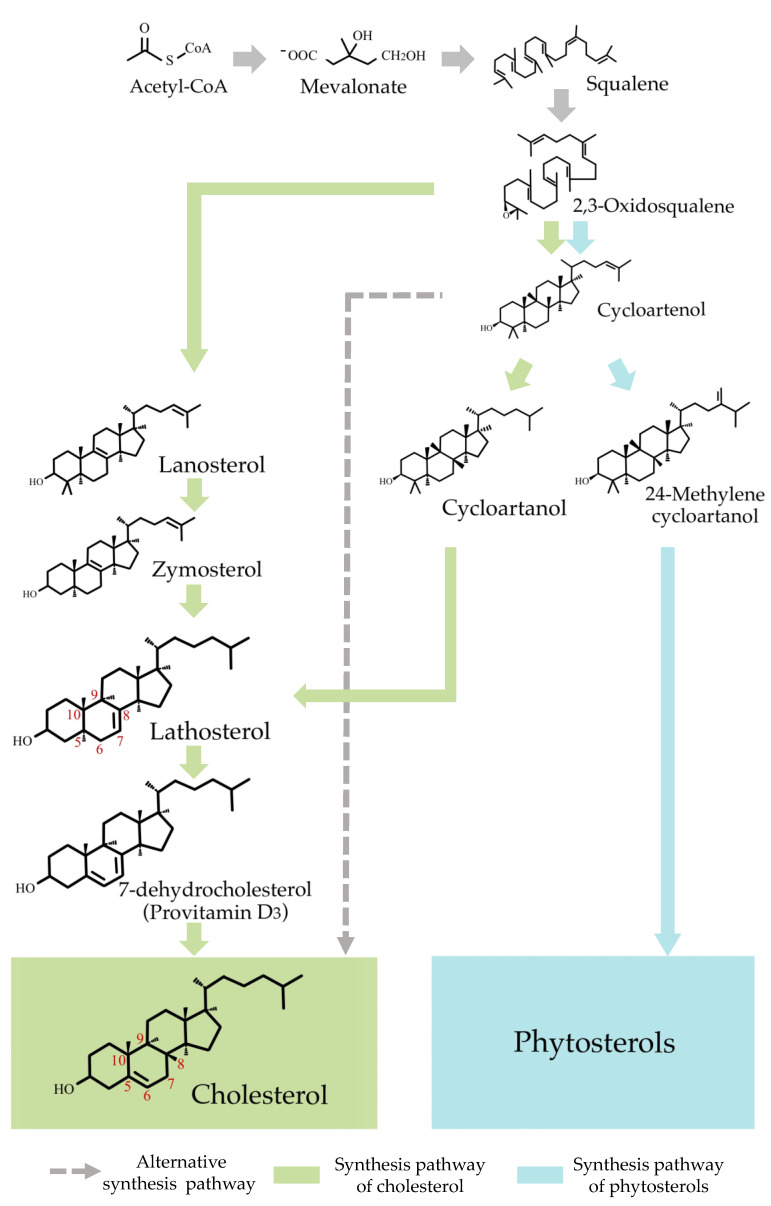
Suggested cholesterol synthesis pathway in algae and plants. This Figure is adapted from Sonawane et al. [21], Yoshida et al. [39], and Belcour et al. [40].

Generally, in animals, 2,3-oxidosqualene is converted to lanosterol through lanosterol synthase, and cholesterol is finally formed via several metabolic compounds [8]. The biosynthetic pathway of cholesterol in plants and algae (Figure 5) is suggested in previous studies [21,39,40]. Yoshida et al. [39] reported that one of the marine algae, *Aurantiochytrium*, would synthesize cholesterol through lanosterol synthase. On the other hand, a cholesterol synthase pathway through cycloartenol synthase was also suggested. Sonawane et al. [21] reported that 2,3-oxidosqualene is converted to cycloartenol, a cholesterol precursor produced by cycloartenol synthase, in plants (tomato and potato). After the conversion of cycloartenol to cycloartanol, lathosterol, a biosynthetic precursor of cholesterol, is sequentially produced. It can therefore be assumed that since the presence of cholesterol was confirmed in both *P. tenera* and perilla seed oil by LC-MS/MS, lathosterol may exist (Figure 5). Moreover, an alternative synthesis pathway of cholesterol has been recently proposed in the red algal model [40].

Until now, there have been very few studies on the content of both lathosterol and cholesterol in plants, but some studies have found that the amount of cholesterol in animals is much higher than that of lathosterol. For example, the ratio of lathosterol to cholesterol in human plasma was found to be approximately 1:1000 [41], and an identical ratio was observed in rat brains [42]. These findings are probably a result of lathosterol being a precursor of cholesterol in cholesterol biosynthesis. Thus, it is possible that even in cases where the quantity of cholesterol is small, trace amounts of lathosterol exist, although detection of such small quantities would be challenging. As such, the presence of lathosterol in perilla seed oil should not be overlooked.

## 3. Material and Methods

### 3.1. Materials and Reagents

Three types of dehydrated algae were purchased from local companies: *P. tenera* (Hanam, Korea)*, S. japonica* (Yeoncheon, Korea)*,* and *U. pinnatifida* (Goheung, Korea). In addition, four perilla seed oil samples (crude oil extracted from *P. frutescens*) were obtained from the local markets of four different Korean cities (Wanju, Seoul, Anyang, and Daejeon). Cholesterol (C8667-5G), 5-α-cholestane (C8003-1G), hexamethyldisilazane (52619-50ML), chlorotrimethylsilane (92360-100ML), and pyrogallol (254002-250G) were purchased from Sigma-Aldrich Chemical Co. (St Louis, MO, USA). Potassium hydroxide (6597-4400), butylated hydroxytoluene (BHT, 2085-1405), and sodium chloride (7548-4400) were purchased from Daejung Chemicals & Metals Co., Ltd. (Siheung, Korea). *N,N*-dimethylformamide (D0722) was purchased from Tokyo Chemical Industry Co., Ltd. (Tokyo, Japan). Lathosterol (700069P-5MG) was purchased from Avanti Polar Lipids, Inc. (Birmingham, AL, USA). Sodium sulfate (83455S0350) was purchased from Junsei Chemical Co., Ltd. (Tokyo, Japan). All solvents used in the analysis were LC-grade and purchased from Fisher Scientific International, Inc. (Waltham, MA, USA).

### 3.2. Extraction

Cholesterol was extracted from algae (*P. tenera, S. japonica,* and *U. pinnatifida*) and perilla seed oil according to the National Laboratory System (NLS) procedure with slight modifications [43]. Each sample (3−5 g) was weighed in an extraction tube before adding distilled water (3 mL) and 6% pyrogallol solution in ethanol (10 mL) and mixing in a vortexer for 2 min. After nitrogen flushing for 1 min, sonication was performed at 0 °C for 10 min, and a 60% potassium hydroxide solution (8 mL) was added before mixing in a vortexer for 2 min. The saponification reaction was then carried out in a shaking water bath at 75 °C for 1 h at 100 rpm. Upon completion, the reaction mixture was cooled in cold water, followed by the addition of 2% sodium chloride solution (20 mL) and an extraction solvent (15 mL, hexane/ethyl acetate = 85:15, *v*/*v*, with 0.1% BHT) before vigorous agitation for 2 min. After phase separation was performed, the upper layer was transferred to a 50 mL volumetric flask through an anhydrous sodium sulfate column. Two more extractions from the lower layer were performed using the extraction solvent (15 mL). Finally, the volumetric flask was filled up to the mark with the extraction solvent. 

### 3.3. Cholesterol Analysis Using Gas Chromatography-Flame Ionization Detector (GC-FID)

For the analysis of cholesterol content, a Younglin M600D gas chromatograph (GC) series (Younglin, Anyang, Korea) equipped with an HP-5 column (30 m × 0.32 mm × 0.25 μm; Agilent Technologies, Santa Clara, CA, USA) and a Younglin 5890 flame ionization detector (FID) were used. The oven temperature was increased from 260 to 280 °C for 10 min. For the verification of the results, a 5-α-cholestane (in *n*-heptane), 0.1 mg/mL, internal standard (IS) was used according to the internal standard method. The relative retention time (RRT = retention time of cholesterol standard/retention time of internal standard) was determined to verify the identity of the cholesterol peaks detected by GC analysis.

Quantification of cholesterol was performed using a cholesterol standard calibration curve obtained at seven concentrations (0.0025, 0.005, 0.01, 0.05, 0.1, and 0.2 mg/mL, in *N,N*-dimethylformamide). The calibration curve was determined by dividing the cholesterol-peak area at each concentration by the peak area of the IS (Table 1). The Y-axis was set to measure the ratio of cholesterol-peak area to IS-peak area, and the X axis was set to measure the concentration of cholesterol. In addition, the limit of detection (LOD) and limit of quantification (LOQ) for cholesterol in GC-FID analysis are listed in Table 1. Derivatization was performed for GC analysis. An organic extract phase (12.5 mL) was evaporated to dryness under nitrogen before adding acetone (3 mL) and *N,N*-dimethylformamide (3 mL). An aliquot (1 mL) of the organic extract dissolved in *N,N*-dimethylformamide was added to hexamethyldisilazane (0.2 mL) and chlorotrimethylsilane (0.1 mL) in a 15 mL test tube and left to react for 15 min.

After reaction completion, distilled water (10 mL) and 5-α-cholestane IS solution (1 mL) were added. After vigorous stirring for 1 min, the upper layer was transferred to the GC vial through an anhydrous sodium sulfate column. The same procedure was followed using a cholesterol standard solution rather than the organic extracts. The cholesterol content was determined using equation (1), where C is the cholesterol concentration (mg/mL), W is the sample weight (g), V_1_ is the volume of extraction solvent used for extraction (mL), V_2_ is the volume of extraction solvent used for concentration (mL), and V_3_ is the volume of *N,N*-dimethylformamide (3 mL).

(1) $$\mathrm{Cholesterol}\;(\mathrm{mg}/100\;g)=C\;\times \frac{V_{3}\times 100}{W\times [V_{2}/V_{1}]}$$

### 3.4. Cholesterol Analysis Using High-Performance Liquid Chromatography-Evaporative Light-Scattering Detector (HPLC-ELSD)

A high-performance liquid chromatographer (HPLC) equipped with an HPLC pump and a degasser (ACME 9000, Younglin, Anyang, Korea) was used with ELSD (ZAM 3000, Schambeck SFD GmbH, Bad Honnef, Germany). To separate cholesterol and lathosterol, a C18 column (Poroshell 120 EC-C18, 4.6 × 75 mm × 2.7 μm, Agilent Technologies, Santa Clara, CA, USA) was used. The mobile phase consisted of acetonitrile and methanol (8:2), and the flow rate was set to 0.7 mL/min. The injection volume was set to 20 μL, and the total run time was 15 min. To quantify cholesterol, an external-standard method was used, and a cholesterol standard calibration curve was obtained with four concentrations (0.005, 0.01, 0.05, and 0.1 mg/mL in methanol). The calibration curve was set up such that the Y-axis measured the cholesterol-peak area, and the X-axis measured the concentration of cholesterol. The LOD and LOQ of HPLC-ELSD are listed in Table 1. The cholesterol content was determined using equation (2), where S is the cholesterol concentration (μg/mL), a is the volume of the solution (mL), and b is the dilution rate.

(2) $$\mathrm{Cholesterol}\;(\mathrm{mg}/100\;g)=S\;\times \frac{a\times b}{sample\;weight\;\left(g\right)}\times 0.1$$

An organic extract phase (12.5 mL) was evaporated to dryness under nitrogen and re-dissolved in methanol (3 mL) before injection.

### 3.5. Cholesterol Analysis Using Liquid Chromatography-Tandem Mass Spectrometry (LC-MS/MS) with Atmospheric Pressure Chemical Ionization (APCI)

A 1290 Infinity II HPLC system (Agilent Technologies, Santa Clara, CA, USA) equipped with a tandem mass spectrometer (QTRAP, 6500, AB SCIEX, Framingham, MA, USA), an electron multiplier detector, and a Poroshell 120 EC-C18 (4.6 × 75 mm, 2.7 µm, Agilent Technologies, Santa Clara, CA, USA) was used [44]. Multiple reaction monitoring (MRM) mode was used for cholesterol identification. The mobile phase consisted of 100% acetonitrile, and 5 µL of the sample was injected. The vacuum pump pressure was set to 2.1 torr, and nitrogen gas was used for fragmentation of precursor ions. Selected characteristic product ions generated from the precursor ions are shown along with collision energies in Table 2. An organic extract phase (12.5 mL) was evaporated to dryness under nitrogen and re-dissolved in methanol (3 mL) before injection.

## 4. Conclusions

The motivation behind this study was to investigate whether cholesterol, a type of lipid that is a well-known component of animal cells, is also present in non-animal organisms. It was determined that low levels of cholesterol are indeed present in some non-animal organisms, and lathosterol (a molecular equivalent) is also present. This was determined through the use of three analytical methods (GC-FID, HPLC-ELSD, and LC-MS/MS), each having different principles of analysis and detection methods. Cholesterol was found to be present in the algae species (*P. tenera*) and in crude seed oil extracted from a plant (*P. frutescens*). In *P. tenera*, different methods of analysis determined similar cholesterol contents (14.6 mg/100 g by GC-FID and 14.2 mg/100 g by HPLC-ELSD). In the perilla seed oil, the cholesterol content was determined by GC-FID analysis to be 0.3–0.5 mg/100 g, but HPLC-ELSD proved unsuccessful at detecting cholesterol, due to the relatively high LOQ. LC-MS/MS, however, was able to confirm the presence of cholesterol in both perilla seed oil and *P. tenera*, suggesting that cholesterol does exist in non-animal organisms.

## Figures and Tables

**Table 1:** Cholesterol contents (mg/100 g) in *Pyropia tenera, Saccharina japonica, Undaria pinnatifida*, and perilla seed oil obtained by gas chromatography-flame ionization detection (GC-FID) and high-performance liquid chromatography-evaporative light-scattering detection (HPLC-ELSD).

Instrument	Range ^a^ (mg/mL)	Calibration Curve ^b^	*R^2^*	LOD ^c^	LOQ ^d^	*Pyropia tenera*	*Saccharina* *japonica*	*Undaria* *pinnatifida*	Perilla Seed Oil
GC-FID	0.0025−0.2	y=9.5761x +0.0266	0.9989	0.10	0.28	14.6	ND	ND	0.3−0.5 ^e^
HPLC-ELSD	0.005−0.1	y=0.9376x +0.0866	0.9985	0.23	0.66	14.2	ND	ND ^f^	TR ^g^

^a^ Range: concentration range of the cholesterol standard used for the calibration curve. ^b^ Calibration curve: calibration curve used to determine cholesterol concentration. ^c^ LOD: Limit of detection refers to the minimum detectable concentration of an analyte (mg/100 g). ^d^ LOQ: Limit of quantification refers to the minimum concentration of an analyte substance that can be expressed in quantitative values (mg/100 g). ^e^ Range of cholesterol amount in the four perilla seed oil samples. ^f^ ND: not detected; ^g^ TR: trace.

**Table 2:** Precursor and product ion pairs, parameters for multiple reaction monitoring (MRM) of standards (cholesterol and lathosterol), *Pyropia tenera*, and perilla seed oil using liquid chromatography-tandem mass spectrometry (LC-MS/MS).

Compounds	MRM Transition		Collision Energy (V)	Peak Area	Relative Ratio ^a^
	Precursor ion *(m*/*z)*	Product ion *(m*/*z)*			
Cholesterol standard	369.5	81.0	65	$4.5\times 10^{6}$	3.3
		95.0	38	$3.7\times 10^{6}$	2.7
		147.0	27	$4.7\times 10^{6}$	3.4
		161.1	23	$1.3\times 10^{7}$	9.4
		189.1	29	$1.9\times 10^{6}$	1.4
		257.1	19	$1.4\times 10^{6}$	1.0
Lathosterol standard	369.5	81.0	65	$1.7\times 10^{4}$	5.8
		95.0	38	$2.1\times 10^{4}$	7.0
		147.0	27	$3.2\times 10^{4}$	10.7
		161.1	23	$1.3\times 10^{5}$	43.2
		189.1	29	$3.0\times 10^{3}$	1.0
		257.1	19	$7.7\times 10^{3}$	2.6
*Pyropia* *tenera*	369.5	81.0	65	$1.1\times 10^{8}$	3.2
		95.0	38	$9.0\times 10^{7}$	2.5
		147.0	27	$1.2\times 10^{8}$	3.3
		161.1	23	$3.3\times 10^{8}$	9.2
		189.1	29	$4.8\times 10^{7}$	1.4
		257.1	19	$3.6\times 10^{7}$	1.0
Perilla seed oil	369.5	81.0	65	$1.1\times 10^{5}$	3.4
		95.0	38	$9.0\times 10^{4}$	2.7
		147.0	27	$1.1\times 10^{5}$	3.4
		161.1	23	$3.3\times 10^{5}$	10.0
		189.1	29	$4.8\times 10^{4}$	1.4
		257.1	19	$3.3\times 10^{4}$	1.0

^a^ Relative ratio: peak area of each product ion/the smallest peak area.

## Data Availability

All relevant data are included in the article.

## Sample Availability

Samples of the compounds are not available from the authors.

## References

[1] Tsoupras A., Lordan R., Zabetakis I. (2018). Inflammation, not Cholesterol, Is a Cause of Chronic Disease. Nutrients.

[2] Mouritsen O.G., Zuckermann M.J. (2004). What’s so special about cholesterol?. Lipids.

[3] Andrade L.D.O. (2016). Understanding the role of cholesterol in cellular biomechanics and regulation of vesicular trafficking: The power of imaging. Biomed. Spectrosc. Imaging.

[4] Sheriff D.S., Ali E.F. (2010). Perspective on plasma membrane cholesterol efflux and spermatozoal function. J. Hum. Reprod. Sci..

[5] Pandit S.A., Bostick D., Berkowitz M.L. (2004). Complexation of Phosphatidylcholine Lipids with Cholesterol. Biophys. J..

[6] Rowlands L.J., Marks A., Sanderson J.M., Law R.V. (2020). 17O NMR spectroscopy as a tool to study hydrogen bonding of cholesterol in lipid bilayers. Chem. Commun..

[7] Clayton P.T., Fernandes J., Saudubray J.-M., van den Berghe G., Walter J.H. (2006). Disorders of bile acid synthesis. Inborn Metabolic Diseases.

[8] Mayes P.A., Botham K.M., Robert K.M., Daryl K.G., Peter A.M., Victor W.R. (2003). Cholesterol synthesis, transport and excretion. Harper’s Illustrated Biochemistry.

[9] Miller W.L. (1988). Molecular Biology of Steroid Hormone Synthesis. Endocr. Rev..

[10] Gylling H. (2014). Clinical utility of serum markers of cholesterol absorption and synthesis. Curr. Opin. Lipidol..

[11] Quintao E., Grundy S.M., Ahrens E. (1971). Effects of dietary cholesterol on the regulation of total body cholesterol in man. J. Lipid Res..

[12] Sweeney J.P., Weihrauch J.L., WeBirauch J.L. (1976). Summary of available data for cholesterol in foods and methods for its determination. C R C Crit. Rev. Food Sci. Nutr..

[13] Dietary Guidelines Advisory Committee (2015). Scientific Report of the 2015 Dietary Guidelines Advisory Committee: Advisory Report to the Secretary of Health and Human Services and the Secretary of Agriculture.

[14] MFDS (2019). Labeling Standards of Foods, Etc.

[15] Ghaleshahi A.Z., Ezzatpanah H., Rajabzadeh G., Ghavami M. (2020). Comparison and analysis characteristics of flax, perilla and basil seed oils cultivated in Iran. J. Food Sci. Technol..

[16] Uma Maheswari M., Reena A. (2017). Phytochemical profiling of the red seaweed, Halymenia dilatata by GC-MS analysis. Int. J. Pharm. Sci. Res..

[17] Schwartz H., Ollilainen V., Piironen V., Lampi A.-M. (2008). Tocopherol, tocotrienol and plant sterol contents of vegetable oils and industrial fats. J. Food Compos. Anal..

[18] Govindan M., Hodge J.D., Brown K.A., Nuñez-Smith M. (1993). Distribution of cholesterol in Caribbean marine algae. Steroids.

[19] Sánchez-Machado D.I., Lopez-Hernandez J., Paseiro-Losada P., López-Cervantes J. (2004). An HPLC method for the quantification of sterols in edible seaweeds. Biomed. Chromatogr..

[20] Behrman E.J., Gopalan V. (2005). Cholesterol and Plants. J. Chem. Educ..

[21] Sonawane P.D., Pollier J., Panda S., Szymanski J., Massalha H., Yona M., Unger T., Malitsky S., Arendt P., Pauwels L. (2016). Plant cholesterol biosynthetic pathway overlaps with phytosterol metabolism. Nat. Plants.

[22] Eller F.J., Moser J.K., Kenar J.A., Taylor S.L. (2010). Extraction and Analysis of Tomato Seed Oil. J. Am. Oil Chem. Soc..

[23] Giera M., Muller C., Bracher F. (2014). Analysis and Experimental Inhibition of Distal Cholesterol Biosynthesis. Chromatographia.

[24] ChemSpider: Cholesterol. http://www.chemspider.com/Chemical-Structure.5775.html.

[25] ChemSpider: Lathosterol. http://www.chemspider.com/Chemical-Structure.59151.html.

[26] Kamenarska Z., Stefanov K., Stancheva R., Dimitrova-Konaklieva S., Popov S. (2006). Comparative investigation on sterols from some Black Sea red algae. Nat. Prod. Res..

[27] Patterson G.W. (1971). The distribution of sterols in algae. Lipids.

[28] Watanabe N., Kimura F., Kojima F., Endo Y., Fujimoto K., Kikuchi Y. (2005). Effect of Sterols in Dietary Fats on Whole Blood Viscosity of Stroke-Prone Spontaneously Hypertensive Rats (SHRSP). J. Oleo Sci..

[29] Torri L., Bondioli P., Folegatti L., Rovellini P., Piochi M., Morini G. (2019). Development of Perilla seed oil and extra virgin olive oil blends for nutritional, oxidative stability and consumer acceptance improvements. Food Chem..

[30] Jeong T.M., Itoh T., Tamura T., Matsumoto T. (1974). Analysis of sterol fractions from twenty vegetable oils. Lipids.

[31] Hojo K., Hakamata H., Kusu F. (2011). Simultaneous determination of serum lathosterol and cholesterol by semi-micro high-performance liquid chromatography with electrochemical detection. J. Chromatogr. B.

[32] Ligor M., Kováčová J., Gadzała-Kopciuch R.M., Studzińska S., Bocian S., Lehotay J., Buszewski B. (2014). Study of RP HPLC Retention Behaviours in Analysis of Carotenoids. Chromatogr..

[33] Choi S.S. (2011). Analysis of polymer additives using LC/MS. Polym. Sci. Tech..

[34] Kang M.J. (2006). Principles and Application of Mass Spectrometry. Polym. Sci. Tech..

[35] Block C., Wynants L., Kelchtermans M., De Boer R., Compernolle F. (2006). Identification of polymer additives by liquid chromatography–mass spectrometry. Polym. Degrad. Stab..

[36] Gachumi G., El-Aneed A. (2017). Mass Spectrometric Approaches for the Analysis of Phytosterols in Biological Samples. J. Agric. Food Chem..

[37] Honda A., Yamashita K., Miyazaki H., Shirai M., Ikegami T., Xu G., Numazawa M., Hara T., Matsuzaki Y. (2008). Highly sensitive analysis of sterol profiles in human serum by LC-ESI-MS/MS. J. Lipid Res..

[38] Menicatti M., Pallecchi M., Bua S., Vullo D., Mannelli L.D.C., Ghelardini C., Carta F., Supuran C.T., Bartolucci G. (2018). Resolution of co-eluting isomers of anti-inflammatory drugs conjugated to carbonic anhydrase inhibitors from plasma in liquid chromatography by energy-resolved tandem mass spectrometry. J. Enzym. Inhib. Med. Chem..

[39] Yoshida M., Ioki M., Matsuura H., Hashimoto A., Kaya K., Nakajima N., Watanabe M.M. (2020). Diverse steroidogenic pathways in the marine alga Aurantiochytrium. Environ. Boil. Fishes.

[40] Belcour A., Girard J., Aite M., Delage L., Trottier C., Marteau C., Leroux C., Dittami S.M., Sauleau P., Corre E. (2020). Inferring Biochemical Reactions and Metabolite Structures to Understand Metabolic Pathway Drift. iScience.

[41] Mackay D.S., Gebauer S.K., Eck P.K., Baer D.J., Jones P.J.H. (2015). Lathosterol-to-cholesterol ratio in serum predicts cholesterol-lowering response to plant sterol consumption in a dual-center, randomized, single-blind placebo-controlled trial. Am. J. Clin. Nutr..

[42] Cibickova L., Radomir H., Stanislav M., Norbert C., Helena Z., Daniel J., Alena T., Eva B., Vladimir P. (2009). The influence of simvastatin, atorvastatin and high-cholesterol diet on acetylcholinesterase activity, amyloid beta and cholesterol synthesis in rat brain. Steroids.

[43] Lee J.H., Na Lee H., Shin J.-A., Chun J.Y., Lee J., Lee K.-T. (2015). Content of Fat-Soluble Nutrients (Cholesterol, Retinol, and α-Tocopherol) in Different Parts of Poultry Meats according to Cooking Method. J. Korean Soc. Food Sci. Nutr..

[44] Fu R., Joseph M. LC/ELSD and LC/MS/MS of Cholesterol and Related Sterols on a Poroshell 120 Column. Application Note 2012; pp. 2–5. http://www.ingenieria-analitica.com/downloads/dl/file/id/2686/product/110/lc_elsd_and_lc_ms_ms_of_cholesterol_and_related_sterols_on_a_poroshell_120_column.pdf.

